# Advances and Translational Challenges in *Toxoplasma gondii* Vaccine Development: From Antigen Discovery to mRNA and One Health Strategies

**DOI:** 10.3390/vetsci13050437

**Published:** 2026-04-30

**Authors:** Abdul Qadeer, Mohamed Tharwat, Muhammad Zahoor Khan, Alexandra Juhasz, Fahad A. Alshanbari

**Affiliations:** 1 School of Medical Sciences, Shandong Xiehe University, Jinan 250109, China; qadeerabdul@sdxiehe.edu.cn; 2 Department of Clinical Sciences, College of Veterinary Medicine, Qassim University, P.O. Box 6622, Buraidah 51452, Saudi Arabia; atieh@qu.edu.sa; 3 College of Agriculture and Biology, Liaocheng University, Liaocheng 252000, China; 4 Institute of Medical Microbiology, Semmelweis University, H-1089 Budapest, Hungary; alexandra.juhasz@lstmed.ac.uk; 5 Tropical Disease Biology, Liverpool School of Tropical Medicine, Liverpool L3 5QA, UK; 6 Department of Medical Biosciences, College of Veterinary Medicine, Qassim University, P.O. Box 6622, Buraidah 51452, Saudi Arabia

**Keywords:** *Toxoplasma gondii*, vaccine development, live-attenuated vaccines, DNA vaccines, mRNA vaccines, immune response, CRISPR/Cas9, One Health, bradyzoite antigens, translational barriers

## Abstract

Toxoplasmosis, caused by the obligate intracellular parasite *T. gondii*, is one of the most prevalent parasitic infections worldwide, affecting approximately one-third of the global population. Despite decades of intensive research, no effective human vaccine exists. The only commercially available vaccine, Toxovax, is restricted to veterinary use in sheep and is unsuitable for human application due to safety concerns. Beyond summarizing the literature, this review offers a critical appraisal of why translation has stalled and where the field should focus next. Live-attenuated vaccines remain the most immunogenic in preclinical models but face significant translational barriers for human use. Key antigenic targets include surface antigens (SAG), dense granule antigens (GRA), rhoptry proteins (ROP), and microneme proteins (MIC). Protective immunity relies critically on Th1-type immune responses characterized by interferon-gamma production. Major obstacles include the parasite’s complex life cycle, strain diversity, and difficulty achieving sterile immunity. Subunit and mRNA-based platforms offer more favorable safety profiles and established clinical precedents, representing the most viable pathway toward a human vaccine. Recent advances in CRISPR/Cas9 gene editing and emerging mRNA vaccine platforms offer promising new directions. This review advances the field in three ways. (i) It prioritizes mRNA and adjuvanted subunit formulations targeting multistage conserved antigens as the most realistic near-term human candidates. (ii) It identifies the limited targeting of bradyzoite-stage biology as a principal, under-addressed gap. (iii) It argues that future development must be differentiated into three complementary One Health goals—prevention of congenital disease in humans, reduction in tissue-cyst burden in livestock, and interruption of environmental transmission by vaccinating cats. In practice, a veterinary-first deployment strategy is the most immediate and impactful pathway to reducing the human and zoonotic burden of toxoplasmosis.

## Simple Summary

This review examines the global effort to develop a vaccine against *Toxoplasma gondii* (*T. gondii*), a common parasite that can harm unborn babies and people with weakened immune systems. Although one vaccine (Toxovax) is already used in sheep to reduce abortion, no safe vaccine exists for humans, and the live sheep vaccine cannot be given to people because of safety concerns. We explain in plain terms which parts of the parasite scientists are targeting, the main kinds of vaccines being tested—including newer types based on the same technology used for COVID-19 vaccines—and why making such a vaccine has proven so difficult. We argue that the fastest way to reduce human infections may not be a human vaccine alone, but also vaccinating cats (which spread the parasite in the environment) and farm animals (which can carry the parasite in meat). Treating animal and human health together—known as the One Health approach—offers a realistic near-term strategy to reduce the global burden of this infection.

## 1. Introduction

**Figure 1:**
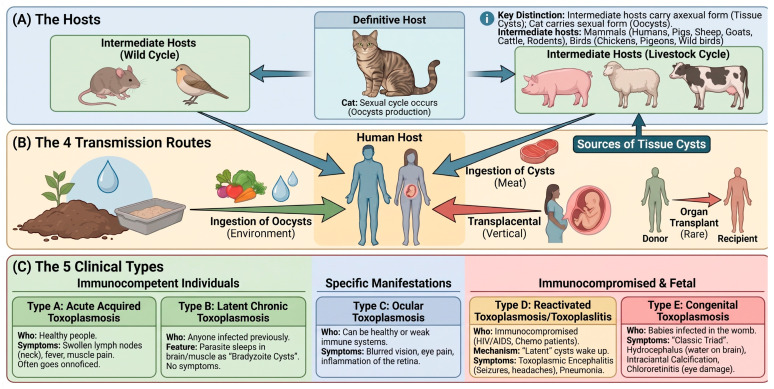
***Toxoplasma gondii* transmission routes and clinical manifestations.** The figure summarizes the definitive (felids) and intermediate hosts, the four principal transmission routes (oocyst ingestion, tissue-cyst-containing meat, organ transplantation, and vertical transmission), and the five clinical forms of toxoplasmosis. Details of each clinical form are described in the main text. Figure generated using Created in BioRender. Khan, M. Z. (2026) https://BioRender.com/12gursm (27 April 2026).

*Toxoplasma gondii* (*T. gondii*) is an obligate intracellular apicomplexan parasite with a remarkable global distribution and the ability to infect virtually all warm-blooded animals [1,2]. With an estimated seroprevalence ranging from 10% to over 80% across different regions worldwide, toxoplasmosis is a significant public health concern affecting approximately 2 billion individuals [3]. The parasite exhibits a complex life cycle involving felids as definitive hosts and numerous intermediate hosts, including humans. Four principal transmission routes have been identified: ingestion of oocysts from contaminated environments; consumption of tissue cysts through undercooked infected meat; organ transplantation; and transplacental transmission [4] (Figure 1).

Beyond direct clinical relevance, *T. gondii* remains highly important from a One Health perspective because livestock infection reflects environmental contamination and may contribute to foodborne exposure risk in humans, as also highlighted by longitudinal serological evidence in beef cattle [5]. Food-producing animals, therefore, act not only as exposure vehicles but also as indicators of the parasite’s environmental circulation, linking environmental, animal, and human health in a way that any rational vaccine strategy must reflect.

In immunocompetent individuals, primary *T. gondii* infection typically results in mild, self-limiting symptoms or remains asymptomatic. However, the parasite establishes a lifelong latent infection by forming tissue cysts containing bradyzoites, primarily in neural and muscular tissues [6]. The clinical significance of toxoplasmosis becomes pronounced in specific high-risk populations, including immunocompromised individuals, such as HIV/AIDS patients and organ transplant recipients, where reactivation of latent infection can cause life-threatening encephalitis. Additionally, primary infection during pregnancy poses severe risks to the developing fetus, potentially resulting in congenital toxoplasmosis characterized by hydrocephalus, intracranial calcifications, chorioretinitis, and even fetal death [7]. Current therapeutic approaches primarily rely on the combination of pyrimethamine and sulfadiazine, which remains the gold-standard treatment [8]. However, these drugs exhibit significant limitations, including severe adverse effects such as bone marrow suppression, Stevens-Johnson syndrome, and hepatotoxicity. Critically, available chemotherapeutic agents are effective only against actively replicating tachyzoites and cannot eliminate encysted bradyzoites, thereby precluding parasitological cure [9]. Furthermore, emerging concerns regarding drug resistance underscore the urgent need for alternative preventive strategies, positioning vaccination as an attractive and potentially superior approach for toxoplasmosis control.

The rationale for developing a vaccine against toxoplasmosis is strongly supported by observations of natural immunity. Immunocompetent individuals who recover from primary infection develop robust, long-lasting protective immunity that prevents reinfection and protects fetuses in subsequent pregnancies [10]. This natural protective response suggests that appropriately designed vaccines could successfully replicate such immunity. However, the convergence of biological, immunological, and translational barriers complicates this goal. Biologically, the parasite’s complex, multistage life cycle and stage-specific antigen expression complicate vaccine design. Immunologically, achieving sterile immunity that eliminates persistent bradyzoite cysts has proven elusive, as current vaccines can reduce disease severity but cannot eliminate chronic infection. Translationally, the most immunogenic platforms (live-attenuated strains) face insurmountable safety and regulatory hurdles for human use, while safer platforms (subunit, mRNA) require optimization to achieve comparable immunogenicity. Furthermore, the absence of clear commercial incentives and defined regulatory pathways has limited investment in human toxoplasmosis vaccine development. Despite this encouraging premise and decades of intensive research, the development of a safe and effective human vaccine against *T. gondii* remains unrealized, making it one of the most challenging objectives in parasitology.

Several comprehensive reviews have summarized *T. gondii* vaccine research in recent years [11,12,13]. The present review is intended to extend rather than duplicate that body of work. Specifically, it fills three gaps that remain incompletely addressed: (i) an integrated critical comparison of platforms with explicit attention to translational readiness rather than preclinical immunogenicity alone; (ii) a conceptual separation of future vaccine objectives into three partially distinct One Health goals; and (iii) explicit discussion of regulatory, clinical-trial, and safety issues (e.g., off-target and reversion risks of CRISPR-attenuated strains) that are typically glossed over in antigen-centric reviews.

## 2. Literature Selection and Search Strategy

This is a narrative review and not a systematic review. The literature was identified through PubMed, Scopus, and Web of Science searches performed up to October 2025 using combinations of the terms “*T. gondii*”, “vaccine”, “mRNA”, “DNA vaccine”, “live-attenuated”, “subunit”, “nanoparticle”, “CRISPR”, “bradyzoite”, “One Health”, and relevant antigen names (SAG, GRA, ROP, MIC, CST1, BAG1). Priority was given to original research articles, systematic reviews, and landmark methodological studies from the last ten years, with earlier seminal work included where historically relevant (e.g., Toxovax). Non-English sources, conference abstracts without full data, book chapters, and reports without clear experimental readouts were generally excluded. This approach is intentionally inclusive rather than exhaustive.

## 3. Current Status of Toxoplasmosis Vaccination

### 3.1. Toxovax: The Only Commercial Vaccine

Toxovax (MSD Animal Health, Wellington, New Zealand), based on the live-attenuated S48 strain of *T. gondii*, represents the sole commercially licensed vaccine against toxoplasmosis [11,12]. This vaccine has been approved for veterinary use in several countries, including those in Europe and New Zealand, primarily to prevent congenital toxoplasmosis and subsequent abortion in sheep. The S48 strain was originally isolated from an aborted lamb in New Zealand and has lost its ability to form tissue cysts following extensive laboratory passage, thereby protecting without establishing persistent infection. The experience with the live S48 strain in sheep clearly demonstrated that protective vaccination against toxoplasmosis is biologically feasible. It also illustrated the practical limitations of live veterinary vaccines in terms of safety, shelf-life, and broader translational applicability [14]. Although effective in reducing abortion rates in vaccinated ewes, Toxovax has significant limitations that prevent its use in humans. These include an extremely short shelf-life of only 10 days, necessitating rapid administration following production, fundamental safety concerns regarding potential reversion to virulence, requirements for continuous cold-chain maintenance presenting logistical challenges for widespread deployment, and critically, the safety profile has not been evaluated in human clinical trials, rendering it inappropriate for human use under current regulatory frameworks [11,12]. Nevertheless, Toxovax provides instructive lessons for human vaccine development. Its success in sheep and goats demonstrates that live infection, even with an attenuated strain, can induce robust protective immunity sufficient to prevent congenital transmission—a key goal for human vaccination. However, the very features that make it effective (replication competence, broad antigen exposure) are precisely what render it unacceptable for human use. The fundamental trade-off between immunogenicity and safety exemplified by Toxovax has driven the field toward non-replicating platforms for human applications, accepting reduced immunogenicity in exchange for superior safety profiles.

### 3.2. Objectives for Human Vaccine Development

The development of vaccines against toxoplasmosis must consider multiple potential target populations with distinct immunological and clinical requirements. For pregnant women and women of childbearing age, the primary objective would be to prevent congenital transmission through preconception immunization or protection against primary infection during pregnancy. For immunocompromised individuals, vaccines may help boost existing immunity and prevent reactivation of latent infections. These human-focused objectives demand a safety-first vaccine profile, prioritizing non-replicating platforms and well-characterized immune correlates. For veterinary applications, vaccines could reduce tissue cyst formation in food-producing animals, thereby diminishing human exposure through consumption of undercooked meat, and in the case of feline vaccines, could potentially reduce environmental contamination with oocysts [13]. Unlike human vaccines, veterinary candidates can more readily accommodate live-attenuated platforms and are evaluated primarily on parasite burden reduction and transmission-blocking efficacy rather than on clinical safety endpoints applicable to immunocompromised or pregnant recipients. An ideal toxoplasmosis vaccine should possess several key characteristics: demonstrated safety across diverse populations, including immunocompromised individuals; ability to induce strong, durable protective immunity; effectiveness against multiple *T. gondii* strains encompassing the major genotypes circulating globally; practicality in terms of storage stability and ease of administration; and cost-effectiveness to enable widespread deployment in endemic regions [9].

Taken together, future vaccine development against *T. gondii* must be separated into at least three partially distinct goals, each requiring a different vaccine profile and measure of success. The first goal—prevention of congenital disease—targets seronegative women of childbearing age and demands a non-replicating platform with a clear human safety record. The second goal—reduction in tissue-cyst burden in food-producing animals—targets the foodborne transmission route, where live-attenuated or adjuvanted platforms may be acceptable under veterinary regulatory frameworks. The third goal—interruption of environmental transmission—targets cats as the sole definitive host, requiring vaccines that specifically prevent oocyst shedding regardless of systemic protection. Recognizing these distinctions is essential for aligning antigen selection, platform choice, and efficacy endpoints with the specific public health impact each goal is intended to achieve. Achieving these objectives depends critically on identifying appropriate antigenic targets that elicit the required immune responses, as described in the following section.

## 4. Antigenic Targets for Vaccine Development

**Figure 2:**
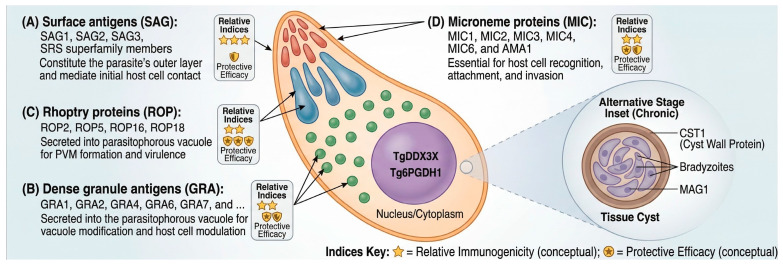
**Antigenic targets for *Toxoplasma gondii* vaccine development.** Schematic representation of: (**A**) surface antigens (SAG); (**B**) dense granule antigens (GRA); (**C**) rhoptry proteins (ROP); and (**D**) microneme proteins (MIC). Created in BioRender. Khan, M. Z. (2026) https://BioRender.com/j32euzx (27 April 2026).

The selection of appropriate antigenic targets represents a critical determinant of vaccine efficacy. Antigens are predominantly derived from specialized secretory organelles unique to apicomplexan parasites: the micronemes, rhoptries, and dense granules [15] (Figure 2).

### 4.1. Surface Antigens (SAG)

SAGs constitute the outermost layer of the parasite and represent the initial point of contact between *T. gondii* and host cells. SAG1 (also known as P30) is the most abundantly expressed and immunodominant surface antigen on tachyzoites, making it the most extensively studied vaccine candidate [16]. This glycosylphosphatidylinositol-anchored protein plays crucial roles in host cell attachment and invasion, and numerous studies have demonstrated that SAG1-based vaccines can elicit both humoral and cellular immune responses, with significant antibody production and IFN-γ secretion. However, SAG1-only vaccines typically provide partial protection, prompting research into multi-antigen formulations. Other surface antigens, including SAG2, SAG3, and members of the SAG1-related sequence (SRS) superfamily, have also been evaluated as vaccine candidates with varying degrees of success [12].

### 4.2. Dense Granule Antigens (GRA)

GRAs are secreted into the parasitophorous vacuole following host cell invasion. The GRA family comprises more than 40 members. GRA1 was among the first cloned [17]; GRA2, GRA4, GRA6, and GRA7 induce robust Th1-biased responses with elevated IFN-γ. Systematic reviews identify GRA4 and GRA7 as particularly protective [18]. Additional members, including GRA14 [19], GRA17, GRA23 [20], GRA15, and GRA24 [21], have also been investigated as vaccine targets, typically in combination with other antigens to enhance protective efficacy.

### 4.3. Rhoptry Proteins (ROP)

Rhoptry organelles contain both bulb proteins (ROPs) and neck proteins (RONs). ROP proteins have garnered significant attention. As introduced above, ROP18, a key virulence factor, has been extensively evaluated, and ROP18-based vaccines have been shown to induce protective immunity, with elevated Th1 cytokine levels and extended survival [22]. Additionally, ROP2, one of the most widely used rhoptry antigens in vaccine research, exhibits strong immunogenicity and has been included in numerous cocktail vaccine formulations. Other rhoptry proteins, including ROP5, ROP8, ROP13, ROP16, and ROP54, have also been evaluated as potential vaccine candidates, and bioinformatic analyses have revealed that certain ROP proteins, such as ROP19, exhibit superior antigenic properties compared to classical candidates like SAG1 [22].

### 4.4. Microneme Proteins (MIC)

MICs are secreted during the initial stages of host cell recognition and attachment, playing essential roles in parasite motility and invasion through adhesive domains that bind host cell receptors. MIC1, MIC2, MIC3, MIC4, and MIC6 have been evaluated as vaccine candidates with promising results, and systematic reviews indicate that MIC3 and its epitopes have been the focus of approximately 57% of microneme-based vaccine studies [23]. These proteins are particularly attractive candidates because they are expressed during the invasive stages of the parasite life cycle, and the combined use of multiple MIC proteins or their incorporation into multi-antigen vaccines has demonstrated enhanced protective efficacy compared to single-antigen approaches. The AMA1 (apical membrane antigen 1), although not strictly a microneme protein, functions in conjunction with RON proteins during invasion and has shown strong immunogenicity in vaccine studies.

### 4.5. Multi-Antigen and Epitope-Based Approaches

Recognition that single-antigen vaccines typically provide only partial protection has driven the development of multi-antigen formulations combining epitopes from different protein families. Cocktail vaccines incorporating antigens from SAG, GRA, ROP, and MIC families have consistently demonstrated superior protective efficacy compared to individual antigen vaccines [24]. For example, DNA vaccines encoding combinations such as TgPF, TgROP16, TgROP18, TgMIC6, and TgCDPK3 have shown enhanced protection against both acute and chronic toxoplasmosis. Multi-epitope vaccines designed using immunoinformatic approaches have emerged as a promising strategy, allowing the rational selection of B-cell and T-cell epitopes from multiple antigens to maximize immunogenicity while minimizing construct size [25].

Critical appraisal of the antigen literature reveals that it is not merely extensive but also lopsided. SAG1, despite high immunodominance, is insufficient on its own, likely because antibody-mediated immunity cannot fully control intracellular parasites. GRA and ROP antigens elicit more robust T-cell responses, with GRA7 and ROP18 demonstrating efficacy across multiple studies. Moving beyond a catalog view, the antigen families can be broadly mapped to distinct objectives: SAG and MIC antigens are best suited to blocking initial invasion and therefore to acute-phase control; GRA and ROP antigens dominate tachyzoite-focused immunity and support reduction in dissemination; and bradyzoite-restricted antigens (notably BAG1 and CST1) are essential for limiting chronic cyst burden and reactivation. Multi-antigen constructs that deliberately combine at least one invasion-associated antigen with one bradyzoite-stage antigen are therefore likely to offer broader life-cycle coverage than tachyzoite-only cocktails.

A major limitation of many current candidates is the predominance of tachyzoite-focused antigen selection. In contrast, stage switching and chronic bradyzoite persistence should be considered more explicitly in rational vaccine design—particularly for long-term protection and reduction in tissue-cyst burden [13]. This issue is not merely theoretical: chronic infection and tissue cyst persistence may have biological consequences in naturally exposed hosts, further supporting the need for vaccine strategies that also target long-term parasite persistence [26].

Bradyzoite-specific antigens such as BAG1 (a stage-conserved heat-shock-like protein) and CST1 (the major cyst wall glycoprotein), therefore, deserve a much more prominent role in vaccine design. Empirically, CST1-displaying VLP vaccines have outperformed tachyzoite-antigen VLPs in reducing chronic cyst burden [27,28], consistent with the view that failure to target bradyzoite biology is a central reason why current vaccines reduce, but do not eliminate, tissue cysts. Achieving anything approaching sterile immunity against *T. gondii* will almost certainly require vaccines that induce CD8^+^ T-cell responses against peptides presented by cyst-harboring cells—a requirement that tachyzoite-dominated antigen panels cannot fulfill.

## 5. Vaccine Platforms and Delivery Systems

### 5.1. Live-Attenuated Vaccines

**Figure 3:**
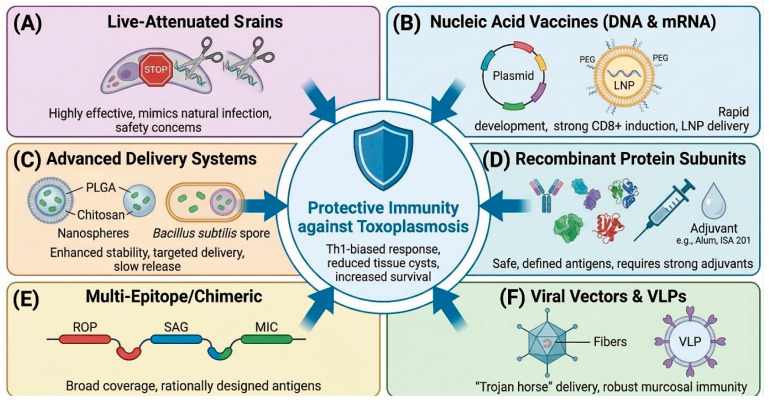
**Vaccine platforms for toxoplasmosis—a comparative overview**. Schematic illustration of the major platforms: (**A**) live-attenuated strains generated by CRISPR/Cas9-mediated gene deletion; (**B**) nucleic-acid vaccines (DNA plasmids and mRNA-LNP); (**C**) nanoparticle-based delivery systems (PLGA, chitosan, calcium phosphate, self-assembling protein nanoparticles); (**D**) recombinant protein subunits adjuvanted to drive Th1-biased immunity; (**E**) multi-epitope or chimeric constructs combining epitopes from ROP, SAG, MIC, and bradyzoite antigens; and (**F**) viral vectors and virus-like particles (VLPs) displaying *T. gondii* antigens. Created in BioRender. Khan, M. Z. (2026) https://BioRender.com/p3nlpcs (27 April 2026).

Live-attenuated vaccines are the most immunogenic approach, inducing robust and durable immunity that mimics natural infection. CRISPR/Cas9 has revolutionized the generation of rationally attenuated strains [29] (Figure 3). Multiple gene-knockout strains have been developed and evaluated as vaccine candidates with remarkable success. The ΔGRA17 mutant provided protection against acute, chronic, and congenital toxoplasmosis in mouse models [30]. The WH3 Δrop18 strain conferred protection against Type I (RH), Type II (ME49), and Chinese 1 strains [31]. Additional promising attenuated strains include ME49Δcdpk3 [32], uracil auxotrophs Δompdc and ΔompdcΔuprt [33], gamma-irradiated attenuated strains (57.1% six-month survival; 99.8% infection reduction) [34], ME49Δ203240 [35], and PruΔUrm1 (complete protection against lethal RH challenge) [36]. A quantitative head-to-head comparison among various vaccines has been provided in Table 1.

A particularly innovative approach involved HAP2-deficient strains that produce non-infectious oocysts [37]. When administered orally to cats, this transmission-blocking vaccine completely prevented oocyst excretion following challenge, highlighting a One Health-compatible route to reducing environmental contamination at the source. Gene-edited attenuated lines may therefore be particularly valuable not only as immunogenic platforms but also as transmission-blocking tools, as shown by experimental vaccination strategies in cats that prevented shedding of wild-type oocysts after challenge [37]. ME49Δgra5 additionally attenuated breast tumor growth and lung metastasis [38]; GRA4-deletion strains showed tumor immunotherapy activity [39].

Despite these advances, live-attenuated *T. gondii* vaccines are unlikely to be acceptable for human use. The principal human-relevant barriers are (1) regulatory unwillingness to administer live apicomplexan parasites to pregnant women or immunocompromised recipients; (2) stringent cold-chain requirements; (3) theoretical risk of reversion to virulence despite defined deletions; and (4) absence of any regulatory precedent for live parasite vaccines in humans. While CRISPR/Cas9 has revolutionized the generation of rationally attenuated strains [29], two specific safety risks are under-discussed and must be addressed before any human application. First, off-target genomic edits are a non-trivial risk; whole-genome sequencing of vaccine strains should be a minimum standard to validate that only intended deletions are present [29]. Second, even defined-deletion strains carry a theoretical risk of reversion to virulence through gene conversion or genomic complementation. This risk is particularly relevant for *T. gondii,* given its sexual cycle in felids, which creates routes for genetic exchange absent in most other vaccine contexts. Therefore, any live-attenuated CRISPR-edited strain intended for human or even widespread veterinary use must be evaluated for genetic stability under conditions that permit sexual recombination [37]. These considerations reinforce the view that non-replicating platforms (mRNA, subunit, VLP) are more readily translatable to human populations.

### 5.2. DNA Vaccines

DNA vaccines have advantages including ease of production, stability, low cost, and ability to induce humoral and cellular responses [19]. Multi-gene constructs outperform single-gene ones: ROP5/ROP7/SAG1 reduced cerebral cyst burden by 76% [40]; ROP21/ROP29 prolonged survival via Th1 polarization [41]; TgGRA28/TgGRA83 with IL-28B adjuvant improved survival and reduced cyst burden [42]. A five-antigen cocktail (ROP5, ROP18, GRA7, GRA15, MIC6) with IL-24 showed superior protection [43]; a six-rhoptry-protein cocktail prolonged survival [44]; TgIST/TgNSM with IL-36γ elicited Th1-biased protection [45]; a GRA35/GRA42/GRA43 cocktail outperformed individuals [46]; a seven-antigen pVAX1-MAF construct significantly prolonged survival [47]. A single-antigen SRS13 DNA vaccine elicited strong IFN-γ and CD8^+^ responses [48]. Despite these encouraging preclinical data, no DNA vaccine has yet been licensed for human use against any pathogen, which, given the field’s decades-long history of strong mouse data and weak human immunogenicity, is itself an important caution for the translation of *T. gondii* DNA vaccines.

### 5.3. Recombinant Protein Subunit Vaccines

Protein subunit vaccines offer defined composition and quality control but have lower immunogenicity and require potent adjuvants [11]. Chimeric proteins SGR (SAG2-GRA1-ROP1) and SMMG (SAG1-MIC1-MAG1-GRA2) show long-lasting protection [49]. Recombinant ROP6 in *S. cerevisiae* yielded 100% survival and significant cyst reduction [50]. The recombinant *T. gondii* DDX3X protein as a vaccine conferred partial protection, evidenced by extended survival after acute challenge with the RH strain (mean survival: 12 days in immunized mice compared with 10 days in controls) and a lower brain cyst burden after chronic PRU infection (410 vs. 616 cysts per brain, approximately 33% lower than controls) [51]. Soluble full-length rSAG1 prolonged survival by ~14.5 days [52]; rTgCalreticulin achieved 100% survival with reduced cyst burden [53]; rTgDDX39 reduced chronic brain-cyst burden by 34% (as reported in the primary study [54]. Multi-antigen protein combinations have also shown promise, as demonstrated by a cocktail vaccine combining TgCDPK3, TgGRA35, and TgROP46, which significantly prolonged survival after acute challenge and reduced brain cyst burden in chronic infection [55]. However, recombinant GRA15 protection was strain-specific, highlighting an important consideration for vaccine design [56].

### 5.4. mRNA Vaccines

The success of COVID-19 mRNA vaccines has stimulated adaptation to parasitic diseases. mRNA vaccines enable rapid development, native antigen expression with correct post-translational processing, and robust immunity [57]. For *T. gondii*: mRNA-LNP encoding TgNTPase-II reduced cyst counts and prolonged survival [58]; a quadrivalent self-amplifying mRNA-LNP (ROP18, TGME49_237490, TGME49_268230, MIC13) achieved 60–80% survival after lethal tachyzoite challenge and 72.5% cyst reduction after oocyst challenge [59]; constructs encoding TGGT1_278620 [60], TG_200 [53], TGGT1_316290 [61], and an optimized ROP6 construct [62] each showed strong Th1-biased immunity and improved survival.

Rather than re-stating the general immunogenicity and safety advantages of mRNA here—which were already established in the COVID-19 program—we note the specific lessons that apply to parasitic vaccines: (i) the mRNA platform’s rapid iterability makes it well matched to multi-antigen *T. gondii* constructs, including stage-switching combinations; (ii) formulated LNPs provide intrinsic Th1-skewing adjuvanticity, which is favorable for *T. gondii* where Th1 responses are protective; (iii) clinical-grade manufacturing and cold-chain logistics are now established, which lowers the translational barrier considerably compared with DNA vaccines; and (iv) crucially, no mRNA vaccine has yet demonstrated efficacy against any parasitic disease in humans, so optimism must be tempered by the recognition that obligate intracellular parasites pose different immunological challenges from viruses. The next step for the field is a human Phase 1 study of a multi-antigen mRNA *T. gondii* vaccine candidate—an achievable, if ambitious, near-term milestone.

### 5.5. Nanoparticle-Based Vaccines

Nanoparticle delivery systems, including PLGA, chitosan, calcium phosphate, and self-assembling protein nanoparticles, have been widely explored [63]. PLGA and chitosan nanospheres loaded with pVAX1-TgIMC1 induced strong immunity with reduced cardiac parasite burden [42]. PLGA-encapsulated TgGAP45 elicited balanced Th1/Th2/Th17 responses [64,65]. Additional formulations—PLGA/chitosan-TgRPS2 [66]; AHACNP-HG hydrogel with MIC6/ROP18 [67]; PLGA-SAG1 with TLR ligands [63]; PLGA-GRA12 vs. GRA7 [68]; alginate-ES antigens [69]; chitosan-SAG1 with propranolol/naltrexone [70,71]—have all shown protective activity.

### 5.6. Virus-like Particle (VLP) Vaccines

VLPs are highly immunogenic platforms that mimic viral structure without genetic material. ISP3-VLP provided complete protection against lethal ME49 challenge [72]. Intranasal VLPs expressing GRA5 [73] or GRA7 reduced brain inflammation and cyst burden [74]. Baculovirus VLPs expressing CST1 or MIC8 elicited protective immunity, with CST1 superior [27]. CST1-based VLPs deserve particular attention as one of the few platforms that specifically target cyst wall biology and therefore the bradyzoite stage. Additional strategies include vaccinia-prime/VLP-boost combinations [75], CST1/ROP18 comparisons [28], heterologous oral ROP4 VLPs [76], and influenza VLPs displaying CST1 to reduce chronic cyst burden [77].

### 5.7. Head-to-Head Platform Comparison

A central question for translation is which platform to prioritize. The following comparative analysis consolidates the qualitative arguments above into a single framework (Table 1).

### 5.8. Novel Adjuvant Systems

Novel adjuvant systems play a crucial role in enhancing vaccine efficacy against *T. gondii*. Colloidal manganese-salt adjuvant with inactivated parasites achieved 50% acute survival and 90.77% cyst reduction [78]. HA201/HA203 adjuvants achieved 50%/70% survival, vs. 10% with the vaccine alone [79]. MIC13/GRA1/SAG1 with Freund’s adjuvant elicited the highest immunity [80]. Nanolipid-encapsulated melatonin and retinoic acid were superior intranasal adjuvants [81,82]. Multistage rBAG1/rGRA1 with chitosan–*Salmonella typhi* porin adjuvant achieved the highest survival [83].

### 5.9. Recombinant Viral Vector and Bacterial Vaccines

Recombinant viral vectors represent an emerging platform for toxoplasmosis vaccination. Intranasal recombinant vaccinia viruses expressing MIC8, AMA1, or RON4 induced 100% survival against lethal ME49 challenges, with MIC8-rVV achieving the greatest cyst reduction [84]. Recombinant vaccinia virus expressing ROP4 induced mucosal and systemic immunity [76,85]. *Bacillus subtilis* spores expressing GRA12 (rBS-GRA12) elicited robust mucosal immunity, resulting in reduced parasite loads [86].

### 5.10. Novel Approaches and Emerging Platforms

Several innovative approaches have emerged in recent research on toxoplasmosis vaccines. Niosome-encapsulated ES antigens boosted immunity, resulting in an 85–90% reduction in parasite load [87]. Exosomes from infected human cells adsorbed to alum reduced the brain cyst burden by 75% [88]. Calcium-mineralization-encapsulated tachyzoites produced thermostable particles that remained stable for >12 months and provided complete protection [89]. The bradyzoite AMA4/RON2L1 moving-junction vaccine specifically reduced chronic cyst burden [90].

## 6. Immunological Mechanisms of Protection

### 6.1. Innate Immune Responses

**Figure 4:**
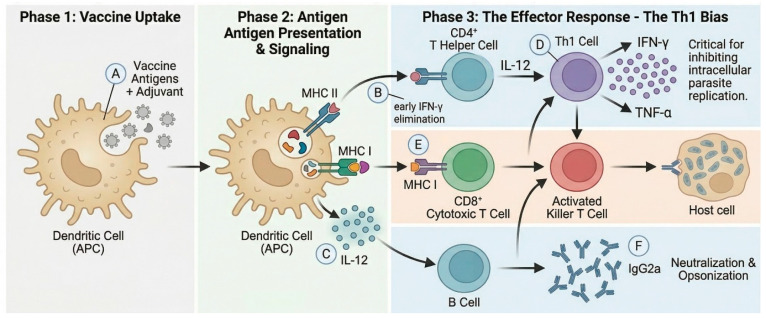
**Immunological mechanisms of protection against *Toxoplasma gondii*.** (**A**) Vaccine uptake: antigen + adjuvant is taken up by antigen-presenting cells (APCs, including dendritic cells). (**B**) MHC-II presentation activates CD4^+^ T helper cells. (**C**) MHC-I cross-presentation activates CD8^+^ cytotoxic T lymphocytes (CTLs). (**D**) IL-12 from dendritic cells drives Th1 polarization and early IFN-γ. (**E**) Th1 cells and CTLs produce high levels of IFN-γ, inhibiting intracellular parasite replication. (**F**) B cells generate IgG2a antibodies that neutralize and opsonize extracellular parasites. The overall protective signature is Th1-polarized with elevated IFN-γ and antigen-specific CD8^+^ T cells. Created in BioRender. Khan, M. Z. (2026) https://BioRender.com/74vg96m (27 April 2026).

The innate immune system plays a critical role in the initial recognition and control of *T. gondii* infection. Parasite-derived molecules are recognized through pattern-recognition receptors, particularly Toll-like receptors (TLRs). TLR11 and TLR12 recognize *T. gondii* profilin in mice, triggering MyD88-dependent signaling and IL-12 production by dendritic cells and macrophages [91]. Critically, humans lack functional TLR11 and TLR12. Hence, mouse-derived efficacy data driven by profilin recognition may not directly translate to humans. Alternative sensing routes in humans include TLR4, TLR2, and intracellular sensors, such as cGAS-STING. Natural killer (NK) cells provide an early source of IFN-γ, and dendritic cells bridge innate and adaptive immunity by processing *T. gondii* antigens and producing IL-12 that directs Th1 differentiation (Figure 4). Neutrophils contribute through phagocytosis and the release of antimicrobial compounds.

### 6.2. Adaptive Immune Responses

Cell-mediated immunity, particularly the Th1 response, is essential for long-term protection. CD4^+^ Th1 cells produce IFN-γ, which controls *T. gondii* by activating infected macrophages, inducing antimicrobial effectors, and coordinating the overall response [92]. IFN-γ activates immunity-related GTPases (IRGs) and guanylate-binding proteins (GBPs) that target and destroy the parasitophorous vacuole. CD8^+^ cytotoxic T lymphocytes are indispensable during chronic infection, eliminating parasite-harboring cells that present *T. gondii* peptides on MHC-I [93]. Humoral immunity provides protection through neutralization, opsonization, and antibody-dependent cellular cytotoxicity; protective responses are associated with Th1-linked IgG (notably IgG2a in mice), while mucosal IgA may limit infection at intestinal entry sites.

### 6.3. Role of Adjuvants

Adjuvant selection critically influences vaccine immunogenicity and the type of response generated. For toxoplasmosis, Th1-promoting adjuvants are preferred. Alum predominantly induces Th2 responses and is therefore suboptimal for *T. gondii* [94]. More appropriate choices include TLR9 agonists (CpG oligodeoxynucleotides), GLA-SE (glucopyranosyl lipid adjuvant-stable emulsion), and cytokine adjuvants such as IL-12, IL-15, IL-21, and GM-CSF. Nanoparticle formulations inherently possess adjuvant properties, owing to enhanced antigen uptake and depot effects [95].

From a translational perspective, several immunological requirements should guide vaccine development: (1) an effective vaccine must induce robust, durable IFN-γ-producing CD8^+^ T-cell responses—this should be the primary immunological endpoint in preclinical evaluation; (2) given the absence of TLR11/12 in humans, candidates showing protection through TLR11-dependent mechanisms in mice may not translate effectively, and studies should incorporate TLR11-knockout mice or alternative readouts; (3) the field lacks standardized immunological correlates of protection—establishing consensus surrogate markers would facilitate cross-study comparison; and (4) mucosal immunity at the intestinal entry point deserves greater attention.

## 7. Challenges in Vaccine Development

### 7.1. Complex Life Cycle and Stage-Specific Antigens

The complex life cycle of *T. gondii*, involving tachyzoites, bradyzoites, and sporozoites with differential antigen expression, complicates vaccine design. Effective vaccines should target antigens expressed across multiple stages [13]. The tachyzoite-to-bradyzoite shift involves significant changes in gene expression; vaccines targeting only tachyzoite antigens may fail to prevent chronic infection.

### 7.2. Genetic Diversity and Strain Variation

*T. gondii* exhibits significant genetic diversity. Three clonal lineages (Types I, II, III) predominate in North America and Europe; more diverse atypical genotypes circulate in South America, Africa, and Asia; Chinese 1 (ToxoDB#9) dominates in East Asia [54]. Antigenic variation raises concerns about cross-protection, and superinfection further complicates vaccine design. Vaccines should incorporate conserved epitopes or multiple strain-specific antigens.

### 7.3. Achievement of Sterile Immunity

Despite numerous candidates demonstrating the ability to extend survival and reduce cyst burden, none has achieved sterile immunity [12]. Why is sterile immunity particularly difficult for *T. gondii*? Several biologically specific reasons combine to make this goal unusually demanding. First, *T. gondii* is an obligate intracellular parasite that rapidly disseminates to immune-privileged tissues such as the brain, eye, and placenta, where immune surveillance is physiologically constrained [6]. Second, bradyzoites within tissue cysts express a restricted antigenic repertoire behind a thick glycoprotein cyst wall that limits access of antibodies and effector cells; they also replicate extremely slowly, which reduces the rate of MHC-I-presented peptide turnover and thereby limits CD8^+^ T-cell surveillance [13]. Third, the parasite actively modulates host cell signaling through ROP and GRA effectors, suppressing IFN-γ responses in the very cells that harbor cysts [89]. Fourth, even immunocompetent humans who clear acute infection naturally remain chronically infected—indicating that nature itself does not deliver sterile immunity. For these reasons, sterile immunity should arguably be abandoned as a realistic vaccine endpoint; instead, clinically meaningful endpoints are reduction in fetal infection, reduction in tissue-cyst burden in food animals, and prevention of oocyst shedding in cats [12,13].

### 7.4. Translation from Animal Models to Humans

The vast majority of *T. gondii* vaccine studies have been conducted in mouse models (BALB/c, C57BL/6). Significant differences exist between murine and human immune responses; mice express TLR11 and TLR12 that recognize *T. gondii* profilin, while humans lack functional equivalents [13]. These differences may result in candidates showing efficacy in mice but failing to translate to humans. Humanized mouse models or alternative animal models may help bridge this gap. More broadly, the limited translation of promising preclinical results into realistic vaccine candidates likely reflects not only antigenic complexity but also the lack of standardized challenge models, harmonized efficacy criteria, and clinically oriented development pipelines [12]. Addressing this will require the field to agree on a small number of reference challenge models (e.g., a type-II ME49 chronic-infection model and an oocyst-challenge model for cats) and on a minimum set of immunological correlates to be reported in all vaccine studies.

### 7.5. Safety Considerations

Safety remains paramount for vaccines intended for pregnant women and immunocompromised individuals. Live-attenuated vaccines, while highly immunogenic, carry inherent risks including potential reversion to virulence, persistence of attenuated parasites, and contraindication in immunocompromised recipients [96]. Even genetically defined attenuated strains require extensive safety evaluation. As discussed in Section 5.1, two specific CRISPR-related risks—off-target genomic edits and reversion via genetic exchange during the sexual cycle in felids—must be addressed through whole-genome sequencing of vaccine strains and prohibition of co-infection with wild-type *T. gondii* during manufacturing. Non-living vaccine platforms offer superior safety profiles but typically require adjuvants.

### 7.6. Regulatory and Clinical-Trial Challenges for Target Populations

A relatively neglected dimension of *T. gondii* vaccine development is the regulatory and clinical-trial pathway. No licensed human toxoplasmosis vaccine currently exists, and consequently, neither a validated regulatory framework nor an established correlate of protection has been formally accepted by major authorities, including the FDA and EMA. This absence of precedent gives rise to several interconnected challenges.

First, classifying live and genetically modified organisms poses an immediate hurdle. In most jurisdictions, live-attenuated and CRISPR-edited *T. gondii* strains would be regulated as genetically modified organisms, requiring mandatory environmental risk assessments alongside standard vaccine safety evaluations—a dual burden that substantially complicates the development timeline. Second, appropriate clinical endpoints remain undefined. Because sterile immunity is widely considered unrealistic, pragmatic endpoints—such as a measurable reduction in seroconversion during pregnancy or a decrease in congenital transmission rates—must be established. No consensus on such endpoints has yet been reached among regulators, industry stakeholders, and academic investigators, representing a significant translational bottleneck.

The two highest-priority human populations—pregnant women (and women of childbearing age) and immunocompromised individuals—present distinct ethical and operational obstacles for clinical trial design. For pregnant women, conventional placebo-controlled trials are ethically constrained. A preconception vaccination strategy with longitudinal follow-up for pregnancy outcomes is a plausible alternative but requires very large cohorts and extended follow-up periods. In high-seroprevalence settings (e.g., Brazil, France), post-licensure real-world evidence studies may be more feasible than traditional Phase 3 trials. For immunocompromised individuals (e.g., those with HIV/AIDS or transplant recipients), challenges include obtaining informed consent when therapeutic alternatives are limited, selecting appropriate comparator arms (e.g., continued chemoprophylaxis versus vaccination), and managing the heightened risk of vaccine failure during active immunosuppression. Addressing these gaps will require early and sustained engagement between vaccine developers and regulatory agencies.

## 8. Host-Specific Vaccine Development

A One Health approach to toxoplasmosis vaccination, targeting multiple points in the transmission cycle, has driven development of host-specific vaccines. In feline hosts, a recombinant GRA12 vaccine adjuvanted with ISA 201 elicited a mixed Th1/Th2 response, reduced oocyst shedding by 20–28%, and extended survival up to 60 days [97]. For porcine applications, a novel inactivated multistage vaccine formulated with tachyzoite and bradyzoite antigens and Quil-A adjuvant induced robust IgG and IFN-γ responses, resulting in ≥95% reduction in muscle-tissue parasite load [98]. The live-attenuated Pru: Δcdpk2 strain induced a protective Th1-biased response in pigs with NK, γδ T, and CD3αβ T-cell expansion [99]. For ovine applications, plant-based SAG1 vaccines fused to plant HSP90 adjuvants reduced pathology and stimulated relevant immunity in lambs [100].

From a One Health perspective, cats and livestock should be considered as separate but complementary vaccine targets. In cats, the objective is transmission-blocking—specifically, prevention of oocyst shedding into the environment, which interrupts the parasite’s sexual cycle at its source and reduces environmental contamination for all downstream hosts, including humans. In livestock (sheep, pigs, cattle), the objective is to reduce the tissue-cyst burden in meat, thereby lowering human exposure to foodborne pathogens when meat is undercooked. These two goals require different vaccine profiles: feline vaccines must block oocyst shedding after challenge (measured as parasites per gram of feces), whereas livestock vaccines must reduce tissue cyst load at slaughter (measured by quantitative PCR or histology of muscle tissue). Conflating the two risks, optimizing vaccines that are suboptimal for either goal, and differentiating them aligns product development with public-health impact and with the differing regulatory frameworks for companion animals versus food-producing species.

## 9. Computational Approaches and In Silico Studies

Computational approaches have accelerated vaccine antigen discovery and rational design. A proteomic screen of the *T. gondii* cytoskeleton, identifying 313 antigenic proteins, 63 of which were recognized by IgM antibodies and 250 by IgG antibodies, providing a prioritized list of candidate antigens for future rational vaccine design [101]. Several bioinformatic analyses have characterized promising antigens, including GRA10, predicted to be immunogenic yet non-allergenic with numerous B- and T-cell epitopes [102], TgROP18 with favorable immunogenic properties and stable structure, rhoptry neck proteins TgRON9 and TgRON10 with confirmed antigenicity and solubility [103], and ROP41 as a non-allergenic immunogenic vaccine candidate [104]. Multi-epitope vaccine design has also advanced significantly, as demonstrated by [25], who designed a vaccine combining GRA6 and MIC3 epitopes predicted to be highly antigenic and to bind strongly to TLR2 and TLR4. Similarly, Majidiani et al. designed TgVax452, an epitope-based vaccine targeting SRS surface proteins predicted to strongly activate TLR4-mediated innate immunity [105]. Li et al. [106] identified novel B-cell and T-cell epitopes from membrane proteins through genome-wide screening, selecting 14 linear B-cell epitopes with high projected population coverage. These computational approaches enable the rational selection of B-cell and T-cell epitopes from multiple antigens to maximize immunogenicity while minimizing construct size, representing a promising strategy for future vaccine development [106].

## 10. Future Perspectives and Emerging Technologies

Several emerging technologies and approaches offer promise for advancing toxoplasmosis vaccine development. The continued application of CRISPR/Cas9 and other gene-editing tools enables the creation of precisely attenuated strains with defined genetic modifications, potentially offering an optimal balance between safety and immunogenicity [31]. High-throughput screening of *T. gondii* genes essential for virulence or survival may identify new targets for attenuation or novel protective antigens. The success of mRNA vaccine platforms against COVID-19 has reinvigorated interest in applying this technology to parasitic diseases, and advances in mRNA stability, lipid nanoparticle formulations, and targeted delivery may enable the development of effective and safe *T. gondii* mRNA vaccines. Self-amplifying mRNA vaccines, capable of producing more antigen per administered dose, represent an attractive evolution of this platform [60].

**Figure 5:**
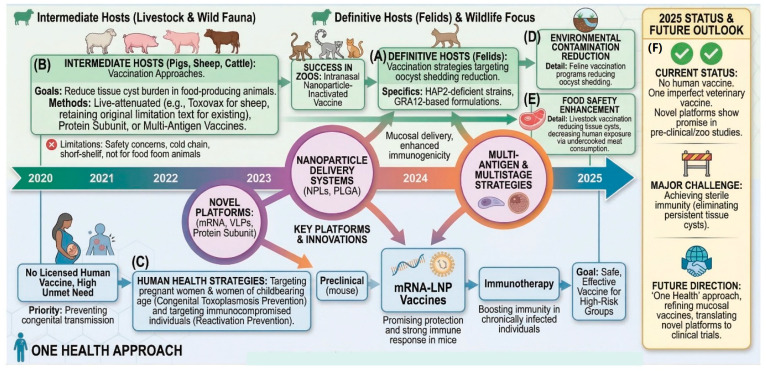
**One health approach to toxoplasmosis control through vaccination.** (**A**) Definitive hosts (felines): Vaccination to prevent oocyst shedding, interrupting the parasite’s life cycle at its source. (**B**) Intermediate hosts (livestock and wild fauna): Vaccination of food-producing animals (pigs, sheep, cattle) using live attenuated (e.g., Toxovax for sheep), protein subunit, or multi-antigen vaccines to reduce tissue cyst burden. Limitations include safety concerns, dependence on the cold chain, and a short shelf life, with restrictions on use in food animals. (**C**) Human health strategies: Targeting pregnant women and women of childbearing age for prevention of congenital toxoplasmosis, and immunocompromised individuals for prevention of reactivation. Despite high unmet need, no licensed human vaccine currently exists. (**D**) Environmental contamination reduction. Feline vaccination programs reduce oocyst shedding into the environment, while livestock vaccination reduces tissue cysts in meat, decreasing human exposure through undercooked consumption. Multi-antigen and multistage strategies are under investigation. (**E**) Food safety enhancement: Vaccination of livestock reduces the tissue cyst burden in meat products, directly enhancing food safety and reducing human exposure risk from consumption of undercooked meat. (**F**) Current status and outlook: No human vaccine is available; only one imperfect veterinary vaccine exists. Novel platforms (mRNA and VLPs) show promise in preclinical studies. Major challenges include achieving sterile immunity. Future directions emphasize immunotherapy, safe vaccines for high-risk groups, and translation of mucosal platforms to clinical trials within an integrated One Health framework. Created in BioRender. Khan, M. Z. (2026) https://BioRender.com/18adzpd (27 April 2026).

Immunoinformatics and reverse vaccinology approaches enable the rational design of multi-epitope vaccines that incorporate optimized combinations of B-cell and T-cell epitopes from multiple antigens. Machine learning and artificial intelligence tools are being applied to predict immunogenic epitopes and optimal vaccine constructs. Prime-boost vaccination strategies, combining different vaccine platforms (e.g., DNA prime followed by protein or VLP boost), have shown enhanced immunogenicity and may help overcome the limitations of individual platforms. An intriguing extension of this approach is a “prime-and-reactivate” strategy for chronic infection, wherein baseline immunity against tachyzoites is first established, followed by controlled immunosuppression to theoretically reactivate dormant bradyzoites, rendering them susceptible to targeting by specific anti-bradyzoite antibodies or therapeutic serum. While experimental attempts to deliberately transition infected animals from chronic to acute states for vaccine efficacy testing remain limited, this concept warrants further investigation as a potential therapeutic vaccination approach. Mucosal vaccination routes, targeting the natural entry point of *T. gondii* through the intestinal mucosa, may provide enhanced protection by inducing local IgA responses in addition to systemic immunity. A one-health approach targeting multiple points in the transmission cycle, including intermediate hosts (food animals) and definitive hosts (cats), may prove more effective than focusing solely on human vaccination. From a veterinary and public health perspective, prioritizing animal vaccination is a more immediately feasible strategy than human vaccination, which faces substantial psychological acceptance barriers and more stringent regulatory requirements. The demonstrated success of Toxovac in sheep provides proof of concept for veterinary applications, and expanding vaccination programs in livestock and companion animals fulfills the One Health objective by reducing the environmental and zoonotic burden at its source. Reducing tissue cyst burden in food animals would decrease human exposure through meat consumption, while vaccines that prevent oocyst shedding in cats would reduce environmental contamination. This integrated One Health strategy—targeting cats, livestock, and humans in parallel—is illustrated in Figure 5, which summarizes the interconnected transmission pathways and intervention points within a One Health framework. A veterinary-first approach offers a pragmatic pathway to protecting human health by diminishing exposure risks rather than relying exclusively on direct human medical intervention, thereby addressing the infection cycle at multiple vulnerable points simultaneously.

## 11. Conclusions

Progress in *T. gondii* vaccine development over the past two decades has been substantial in antigen discovery, immunological understanding, and platform diversity, yet disproportionately modest in the number of licensed human products. The central unresolved tension is the trade-off between safety and immunogenicity: live replicating platforms achieve broad multistage immunity but cannot be used in pregnant women or immunocompromised individuals, while non-replicating platforms are safe but have historically under-targeted the bradyzoite biology underlying chronic infection and reactivation. Resolving this tension requires three shifts. First, deliberately include bradyzoite-stage antigens (BAG1, CST1, MAG1, SAG2Y, ENO1, LDH2) in multi-antigen constructs rather than relying solely on tachyzoite-biased selection. Second, converge on mRNA and adjuvanted subunit platforms as the realistic near-term route for human vaccination, supported by standardized challenge models and harmonized efficacy endpoints. Third, explicitly adopt a differentiated One Health strategy in which veterinary-first deployment—vaccinating cats to block environmental transmission and livestock to reduce the foodborne burden—proceeds in parallel with, not in deference to, human vaccine development. In our view, a veterinary-first, mRNA-anchored, bradyzoite-aware strategy represents the most defensible path from preclinical promise to measurable reduction in global toxoplasmosis burden. Achieving this will require regulatory agencies to accept robust efficacy data from at least two animal species (e.g., mouse and pig, or mouse and cat) against multiple *T. gondii* strains; international harmonization of these preclinical requirements would substantially accelerate translation.

## Figures and Tables

**Table 1:** Comparative assessment of major vaccine platforms for *T. gondii*.

Platform	Immunogenicity	Safety	Stage-Specific Protection	Translational Readiness	Key Limitation
Live-attenuated	Very high	Low (reversion risk)	Broad (acute + chronic)	Veterinary only	Not acceptable for human use
DNA	Moderate	High	Limited (mostly acute)	Low (no licensed human DNA vaccine)	Weak human immunogenicity
Recombinant subunit	Low–moderate (adjuvant-dependent)	High	Acute; limited chronic	Moderate–high	Requires potent Th1 adjuvants
mRNA-LNP	High	High (clinically proven)	Acute; emerging chronic	High	Untested against parasites in humans
Virus-like particle (VLP)	High	High	Acute; some chronic (e.g., CST1-VLP)	Moderate	Manufacturing complexity
Nanoparticle-delivered subunit	Moderate–high	High	Acute; variable chronic	Moderate	Formulation cost/complexity
Viral-vectored	High	Moderate	Acute + mucosal	Moderate	Anti-vector immunity

## Data Availability

No new data were generated for this review article. Data sharing is not applicable to this article.
